# Hamate’s coronal fracture: diagnostic and therapeutic approaches based on a long-term follow-up

**DOI:** 10.3205/iprs000131

**Published:** 2019-03-29

**Authors:** Christian Eder, Ariane Scheller, Nina Schwab, Björn Dirk Krapohl

**Affiliations:** 1 Centre for Musculoskeletal Surgery, Charité – Medical University, Campus Virchow Clinic, Berlin, Germany; 2 Healthcare Center Meviva, Berlin, Germany; 3 Department of Cranio-maxillofacial, Plastic, and Reconstructive Surgery, Carl-Thiem-Klinikum, Cottbus, Germany

**Keywords:** hamate fracture, carpal fractures, Ebraheim’s classification

## Abstract

Hamate fractures are commonly divided into hook fractures and body fractures. The coronal fractures as a special form of hamate’s body fracture are very rare injuries. Because of unspecific clinical findings and the mostly inconclusive x-ray imaging, these fractures are frequently overseen or misdiagnosed. This leads to further complications like secondary arthritis, persisting pain, and functional deficits in patient’s wrist mobility. In our study, a collocation of coronal hamate fractures is analyzed and evaluated with respect to functional outcome after operative treatment and compared to the literature. Furthermore, we compare the strategies for diagnosis and treatment in our clinical center with those presented in the literature.

Our standard in the initial diagnostic process is to obtain radiographs in an anterior-posterior, lateral, and 30° oblique view of the wrist. For further diagnosis and preoperative planning, a CT scan of the wrist is obligatory. Due to the high occurrence of comorbidities (especially CMC dislocations) all patients in our cohort obtained operative treatment.

In long-term post-operative evaluation, we present the following results:

The Manchester-Modified Disability of the Shoulder, Arm and Hand Score (M^2^ DASH) imposed with an average of 26.22 points (MD=22/ SD=11.31/MIN=18/MAX=52). None of the re-evaluated patients sorrowed for severe pain in rest. Four patients stated pain (ranging from 3 to 5 on numeric analogue scale) after heavy burden (e.g. boxing, weight lifting). In exploring the range of motion of the operated hand the following results are obtained: dorsal extension: average 83.33° (MD=85°/SD=3.54°/MIN=75°/MAX=85°), flexion: average 77.78° (MD=80°/SD=4.41°/MIN=70°/MAX=80°). Additionally, a performance testing was conducted: fist clenching sign: complete without pain in 100%, pinch grip: complete in 77.78%, opposition digitus manus I–V complete in 66.67%.

The conservative treatment is not recommended (especially shown in the here presented “add” case with a misdiagnosed fracture). The open approach has its advantages compared to a closed operative procedure and should always be intraoperatively considered as an operative expansion.

## Zusammenfassung

Hamatumfrakturen werden in Hamulus-ossis-hamati- und Corpusfrakturen unterteilt. Die coronare Os hamatum Fraktur ist, als Sonderform der Corpusfraktur, eine sehr seltene Verletzung. Aufgrund der unspezifischen klinischen Symptomatik und unsicheren projektionsradiografischen Diagnostik wird dieser Frakturtyp in der Primärdiagnostik häufig übersehen oder fehldiagnostiziert. Dies führt zu möglichen Folgekomplikationen, wie posttraumatischer Arthrose, persistierender Schmerzzustände und funktioneller Defizite.

In der vorliegenden Studie werden die an unserem Zentrum behandelten coronaren Hamatumfrakturen zusammengestellt, anhand des funktionellen Ergebnisses nach operativer Versorgung ausgewertet und mit der aktuellen Literatur verglichen. Weiterhin wird der diagnostische und therapeutische Algorithmus unseres Zentrums dem publizierten Standard gegenübergestellt.

Standdarddiagnostikum sind Röntgenbilder des Handgelenks in drei Ebenen (anterior-posterior, streng seitlich, 30° schräg – Aufnahme). Zur weiteren Diagnostik und insbesondere präoperativen Planung ist die Computertomografie des Handgelenks obligat. Aufgrund des häufigen Vorhandenseins von Begleitverletzungen (insbesondere carpometacapale Dislokationen) wurden alle Patienten operativ therapiert. Im Rahmen der klinischen Langzeitevaluation zeigten sich folgende Resultate: Der Manchester-Modified Disability of the Shoulder, Arm and Hand Score (M^2^ Dash) lag im Durschnitt bei 26,22 Punkten (MD=22 / SD=11,31 / MIN=18 / MAX=52). Keiner der nachuntersuchten Patienten klagte über starke Ruheschmerzen. Vier Patienten gaben Schmerzen von 3–5 auf der numerischen Analogskala nach stärkerer Belastung (beispielsweise Boxen, Anheben von Lasten) an. Bezüglich des Range of Motion zeigten sich folgende Ergebnisse: Dosalextension: Durchschnitt 83,33° (MD=85° / SD=3,54° / MIN=75° / MAX=85°), Flexion: Durchschnitt 77,78° (MD=80° / SD=4,41° / MIN=70° / MAX=80°). In der Funktionsprüfung zeigten sich: Faustschluss vollständig und schmerzfrei in 100%, Pinzettengriff vollständig in 77,78% und Opposition Daumen–Kleinfinger vollständig in 66,67%.

Insbesondere aufgrund des hier gezeigten fehldiagnostizierten Falles („add“ case) kann das konservative Vorgehen nicht empfohlen werden. Im Vergleich mit einem geschlossenen operativen Verfahren zeigt das offene Vorgehen seine Vorteile und sollte intraoperativ als mögliche Erweiterung in Erwägung gezogen werden.

## Introduction

The hamate bone consists of two different anatomic parts: the hook and the body. Proximally it forms an articulation with the triquetrum and distally it articulates with the fourth and fifth metacarpals (hamato-metacarpal articulation, CMC: carpometacarpal articulation). Fractures of the hamate bone are stated with 2–4% of all carpal injuries [1], [2], [3], [4], [5] and were classified firstly by Milch et al. [6]. He described type 1 fractures as body fractures with fracture line passing either the hook ulnarly or radially and type 2 as hook fractures [6]. 

Within Milch’s classification, the coronal fracture of the body has not been considered. In further studies, other classifications have been introduced, such as Cain’s or Ebraheim’s classification with a more specific interest on body fractures of the hamate [7], [8], [9], [10]. 

The coronal fracture in general appears mostly after punch injuries with a radial-abducted and approximately 10° flexed hand [11], [12] against an unyielding object [2], [4], [5], [7], [8], [9], [10], [11], [12], [13], [14], [15], [16], [17], [18], [19], [20], [21], [22]. 

The diagnostic is intricate because of few clinical findings and often inconclusive initial x-ray observances [2], [5], [6], [7], [8], [9], [10], [11], [14], [16], [19], [20], [23], [24], [25], [26]. 

Moreover, even the treatment of coronal hamate-fractures is still unclear nowadays [2], [5], [16]. Radiological and clinical findings can lead either to a conservative or operative (open versus closed) treatment. 

This paper aims to show a more sensitive diagnostic approach as well as different surgical options in treating coronal fractures of the hamate. We present the largest case series ever published and performed a long-term follow-up of the patients treated in our centre, to create a more detailed view on this specific carpal injury.

## Material and methods

From 2009 through 2014, 19 cases of coronal hamate fractures were diagnosed in the Centre for Musculoskeletal Surgery of Charité Berlin, Campus Virchow Clinic. Initially patients were examined in the emergency unit and transferred to our specific outpatient clinic for hand surgery for further diagnostic and treatment afterwards. 

Retrospectively, patients’ medical recordings have been reviewed. Therefore, age and gender, previous medical history, trauma history, accompanied injuries, operative technique (approaches, operation time, complications), pre- and postoperative radiological images, outcome up to three months after operation, and healing rates were analyzed and summarized under short-term evaluation. 

Furthermore, a long-term evaluation was performed in nine patients. 

The long-term evaluation consisted of two parts to create the best and most detailed view of the long-term results possible. On the one hand, the subjective patient’s opinion concerning their individual reconvalescence was taken by offering the Manchester-Modified Disability of the Arm, Shoulder and Hand questionnaire (M² DASH) [27]. On the other hand, x-ray analysis, objectification of grip strength regain via investigation by hand-held dynamometer (Jamar^®^) [28], and a clinical examination with special regard to range of motion, sensory deficiencies, pain, and strength were conducted. The physical examination was done by the same doctor in all cases with the use of a goniometer and a standardized evaluation protocol. The M² DASH itself includes three thematic subunits:

- 11 activities of daily living (e.g. writing, washing hair, putting on a pullover etc.); scaling 1 point (no difficulties) up to 5 points (not even possible)
- 3 symptoms: paresthesia, weakness, stiffness; scaling 1 point (not present) up to 5 points (extreme)
- 4 psychosocial aspects (e.g. social interference, vocational interference, sleeping disorders, depressive mood); scaling 1 point (not present) up to 5 points (extreme)

Therefore, the highest attainable, and most negative, amount is 80 points. The lowest score is 18 points, showing the best results in all subunits. 

As a retrospective study, an evaluation of existing radiographic imaging during the clinical follow-up (usually up to three months after initial treatment including removal of temporary arthrodesis) was obtained. 

It was relinquished to generate new radiological images in long-term evaluation due to none or just moderate clinical conspicuousness; the radiation exposure would not have been ethically and medically justifiable except in cases with clinical anomalies.

SPSS v24.0 (SPSS Inc., Chicago, Illinois) has been used to create all descriptive statistics including average, the mean (MD), standard deviation (SD), minimum (MIN) and maximum (MAX) and percentage.

Relevant literature and publications were identified using the internet database MEDLINE (including OLDMEDLINE). Further references were found manually.

### Patients’ data 

The average age of the 19 patients initially operated was 25.37 years (MD=22/SD=7.6/MIN=16/MAX=45) when fracturing the hamate bone. The cohort includes 18 male (94.74%) and one female (5.26%).

All of the patients were initially referred to our emergency unit. After initial x-ray diagnosis and computer tomographic scan, the surgery was performed in the Department for Musculoskeletal Surgery of Charité – Medical University of Berlin. 

For long-term re-evaluation one patient had to be excluded due to being younger than 18 years. Furthermore, three patients were lost in follow-up and five patients denied taking part in another clinical examination since they were not experiencing any symptoms or problems. Additionally, one case (external misdiagnosing) came to our hospital with an already malpositioned healed hamate including chronic carpometacarpal subluxations and secondary arthrosis which led to exclusion from long-term reevaluation. This patient got an arthrodesis wherefore ROM and grip strength examination as well as performance testing would have been distorted. So, in conclusion nine patients were clinically examined, again with a mean follow-up of 33.67 months (MIN=14/MAX=54).

In all but one patient evaluated in long-term outcome the fracture affected the dominant hand (88.89%). In seven cases (77.78%) the right hand was fractured, the left one in three cases.

## Results

### Short-term evaluation

In short-term analysis, the data of 19 patients with coronal fracture of hamate’s body were listed. Three of them suffered from externally overseen carpal injury. 

**Figure 1:**
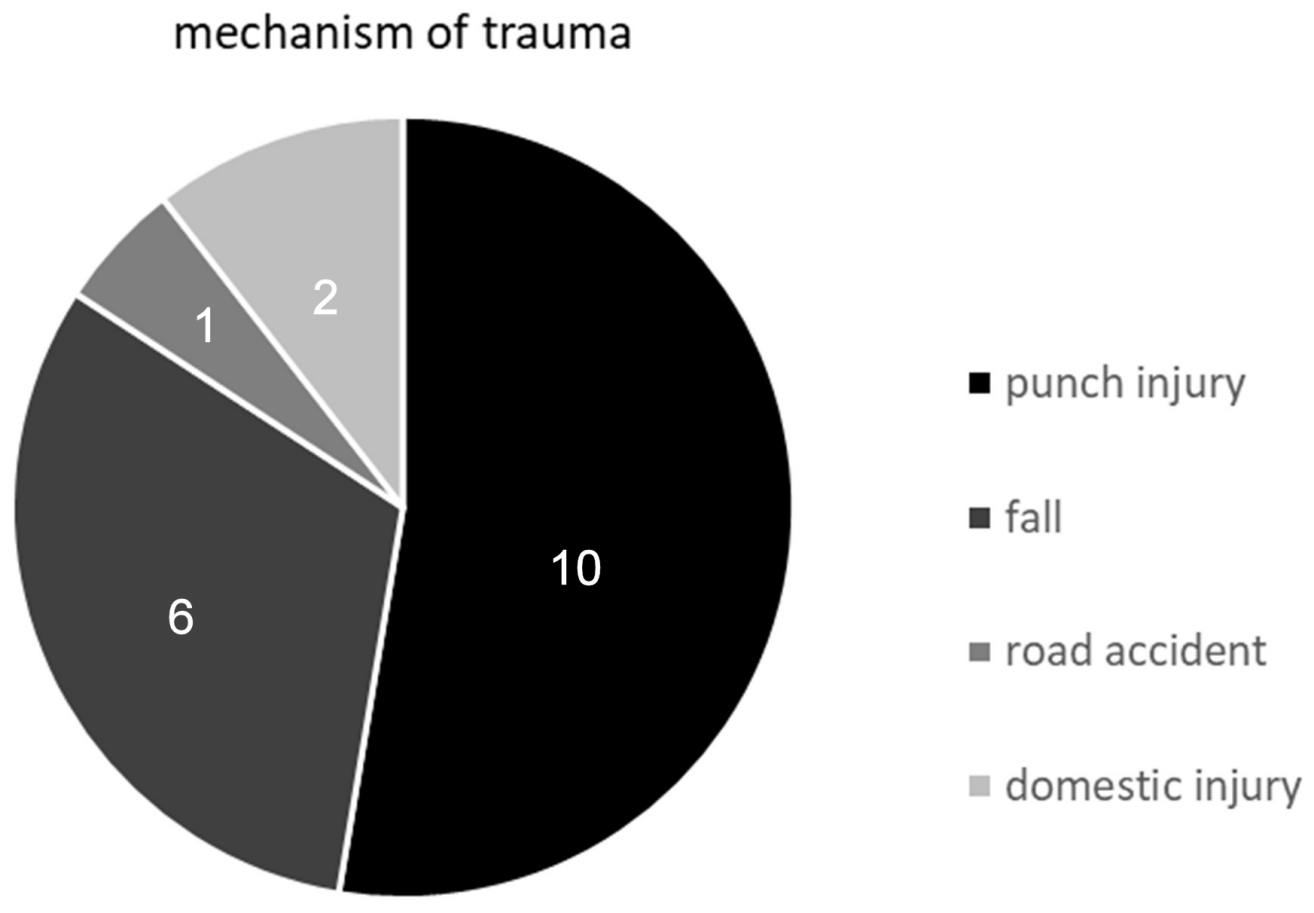
Mechanisms of trauma leading to a coronal fracture of the hamate in our cohort

Mechanisms of trauma based on anamnestic inquiry (Figure 1 (Fig. 1)):

- 10 cases of punch-injury
- 6 cases of fall
- 1 case of traffic accident
- 2 cases of domestic injury

A post-operative immobilization-time of 5.31 weeks in average (SD=0.75) was indicated. Decision about length of immobilization was made intraoperatively concerning the grade of stability in combination with the anticipated compliance of the patient towards postoperative treatment. At the end of regular therapy and after the last indicated X-ray images (or CT if plain radiograph findings were suspicious) in average 3 months post-surgery, 18 patients (80.95%) imposed with bony healing of hamate’s fracture in physiological anatomic position. One patient (initially misdiagnosed externally six weeks ago and malpositioned healing of hamate including secondary arthrosis at initial presentation) had a satisfying arthrodesis situation. 

Table 1 (Tab. 1) visualizes patients’ co-injuries, treatment of accompanied injuries, and the type of osteosynthesis (including specific material) of hamate body chosen in the individual content.

Dislocations of at least one CMC joint were present in 18 of our 19 cases (94.74%). 12 of them had dislocations in more than one CMC (most commonly MC IV combined with MC V dislocations) (63.16%). Furthermore, bony avulsion of ligaments imposed in 42.11% (eight cases): six patients ruptured the dorsal carpometacarpal ligaments, whereas two patients had an avulsion in the dorsal intercarpal ligament fixing the hamate onto the capitate. 

Additional fractures in combination with the hamate fracture:

- metacarpal III: 6 cases (31.58%)
- metacarpal IV: 12 cases (63.16%)
- metacarpal V: 2 cases (10.53%)
- capitate: 4 cases (21.05%)

None of the here presented patients had an isolated coronal fracture of the hamate body without any accompanied injuries.

The specific therapeutic approach for the co-injuries is shown in Table 1 (Tab. 1) as well. The decision whether dislocations in the carpometacarpal joints required an arthrodesis or not, was made intraoperatively; the stability was evaluated after correct osteosynthesis of the hamate bone and, if fractured, the metacarpal bones. 

**Figure 2:**
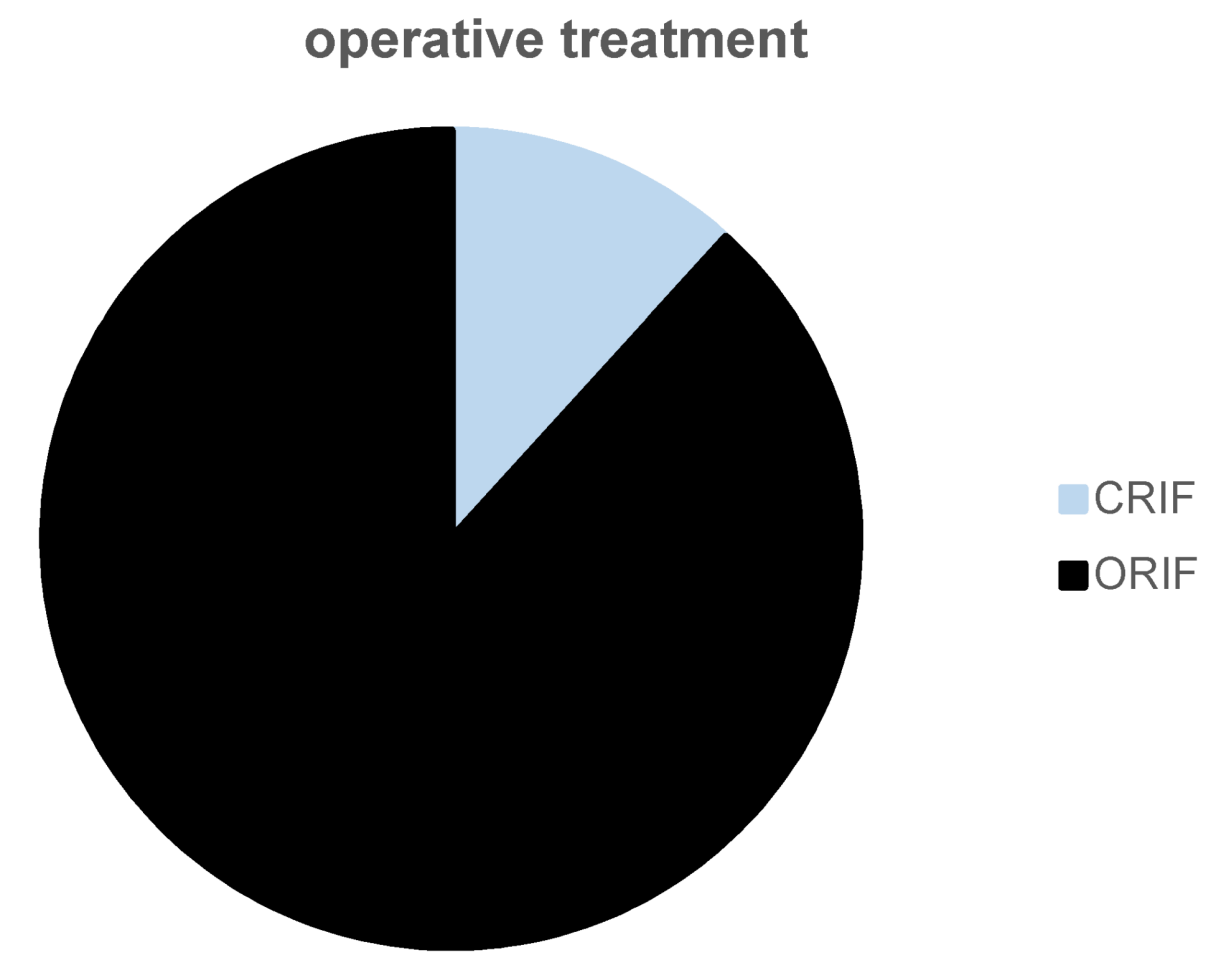
Distribution of open versus closed surgery in the here presented cohort

**Figure 3:**
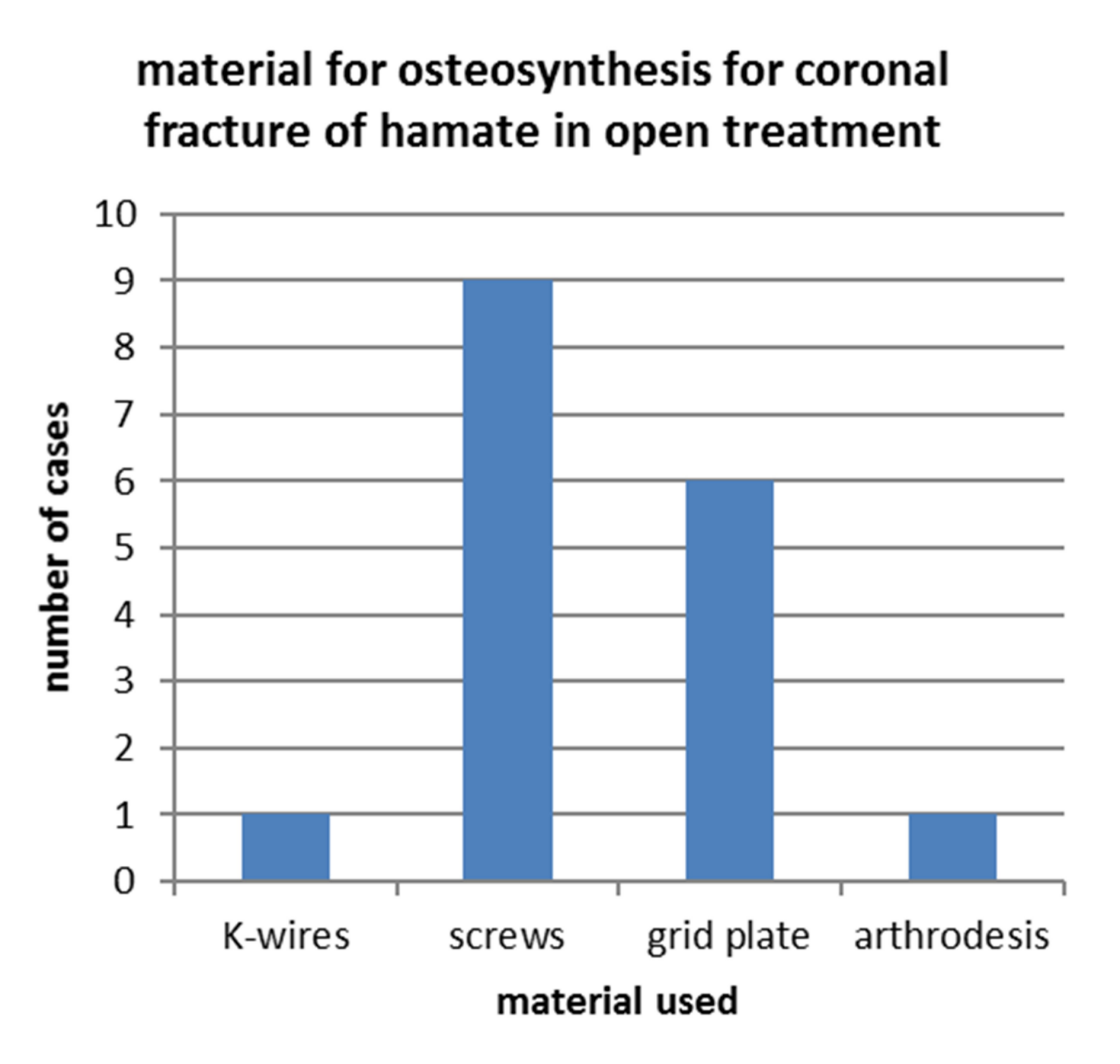
Distribution of different material for osteosynthesis in the subpopulation treated with ORIF

Figure 2 (Fig. 2) and Figure 3 (Fig. 3) show the therapeutic concept in the here presented case series addressing the hamate fracture itself, visualizing the distribution of closed versus open reduction and the material used for open approach respectively.

All 19 patients were treated surgically, due to the following reasons: severity of fracturing implicating no success for conservative approach, unstable situation in all cases (CMC dislocation, ligamentous rupture, co-injuries), delay in three cases because of externally misdiagnosis, demand of all patients for a fast recovery and return to work/physical activity.

17 patients were treated with open reduction (fractures type A or B in Ebraheim’s classification), whereas in two cases fracture repositioning was done via closed reduction and K-wire osteosynthesis (Figure 2 (Fig. 2)). The group of patients with open reduction and internal fixation got the osteosynthesis in (Figure 3 (Fig. 3)):

- 1 case with K-wire OS
- 9 cases with OS via screws
- 6 cases with OS via grid plate
- 1 case with misdiagnosed hamate fracture externally and secondary arthrosis including chronic subluxation of MC bones as well as malpositioned healing of hamate, where arthrodesis was the only option left

Main causes for using different material were the number and sizes of fragments, the accompanied injuries and the grade of stability anticipated. A CT scan preoperatively plainly simplifies the preparation for operative procedure and is even necessary to make clear whether an open approach is needed or a CRIF (closed reduction and internal fixation) should be considered. 

**Figure 4:**
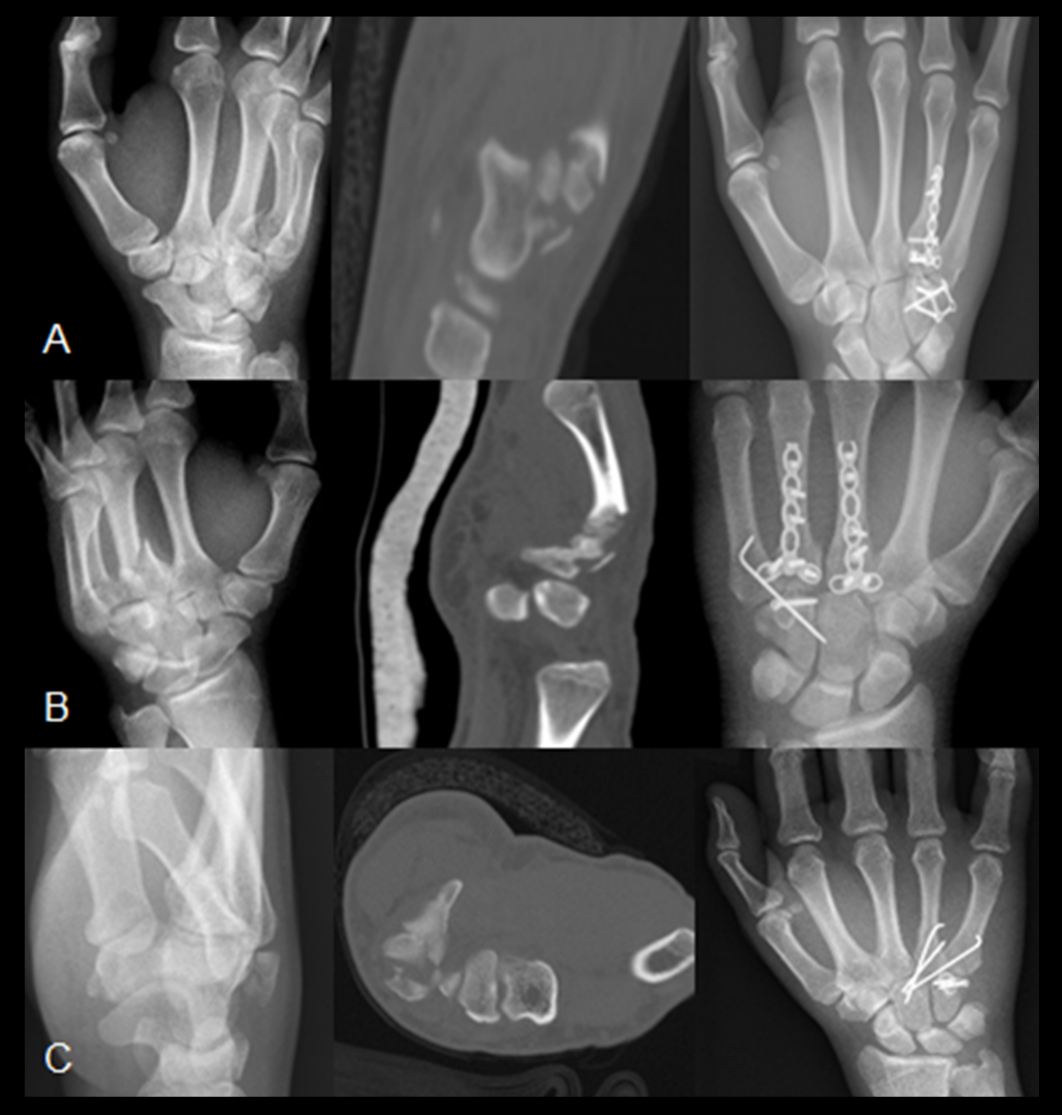
Three patients with coronal hamate’s fracture (A – grid plate OS, B – K-wire and screw OS, C – screw OS). From left to right: preoperative x-ray, preoperative CT scan, postoperative x-ray

Figure 4 (Fig. 4) shows three radiological series, consisting of one exemplary pre-operative x-ray image, pre-operative CT image, and the post-operative final x-ray. Patient A was treated with a grid plate, patient B via K-wire osteosynthesis and patient C got a screw osteosynthesis of the hamate body.

Immobilization (casting in short thumb cast) was conducted for either fore, five or six weeks. The length depended on the accompanied injuries, the grade of instability and the patient’s compliance.

The dissolution of arthrodesis in CMC joints was timed for five to six weeks after initial operation. All patients got x-ray imaging for final decision whether the ulnar carpal region was considered stable and allowed removing of the arthrodesis. The last and essential step in regular treatment was the referral to specialized occupational therapists and physiotherapists to regain best functional outcome possible. 

### Long-term evaluation

In long-term evaluation, nine patients could be included. 

For subjectively measuring patient’s satisfaction with the treatment out-come, the M² DASH was used. 

The Manchester-Modified Disability of the Shoulder, Arm and Hand Score (M² Dash) imposed with an average of 26.22 points (MD=22/SD=11.31/MIN=18/MAX=52). Three patients got 18 points, showing the best results possible; whereas one patient had a poor outcome with 52 points in the M² DASH. This patient missed the date for removal of the arthrodesis K-wires (actually five weeks after definitive care) that may contribute to the unsatisfying result.

None of the re-evaluated patients sorrowed for severe pain in rest. One patient complained about rest-pain seldomly and one after mild activities. Four patients suffered from pain (ranging from three to five on NAS) after heavy burden (e.g. boxing, weight lifting). There were no clinical or radiological reasons visible for these pain episodes, except in the patient with still enclosed arthrodesis K-wires. 

Additionally, one patient noticed dysaesthesia after heavy burden in the dorsal of CMC IV. 

By exploring the range of motion of the operated hand, these are the results:

- dorsal extension: average 83.33°
  (MD=85/SD=3.54/MIN=75/MAX=85)
- flexion: average 77.78°
  (MD=80/SD=4.41/MIN=70/MAX=80)
- ulnar deviation: full range of motion in 100%
- radial deviation: full range of motion in 100%

Additionally, a performance testing was conducted:

- fist clenching: complete without pain in 100%
- pinch gripcomplete in 77.78%, gap of 0.2 cm in 1 case, gap of 0.5 cm in 1 case, pain free in 100%
- opposition digitus manus I–V complete in 66.67%, gap of 0.1 cm in 1 case, gap of 0.5 cm in 2 cases

**Figure 5:**
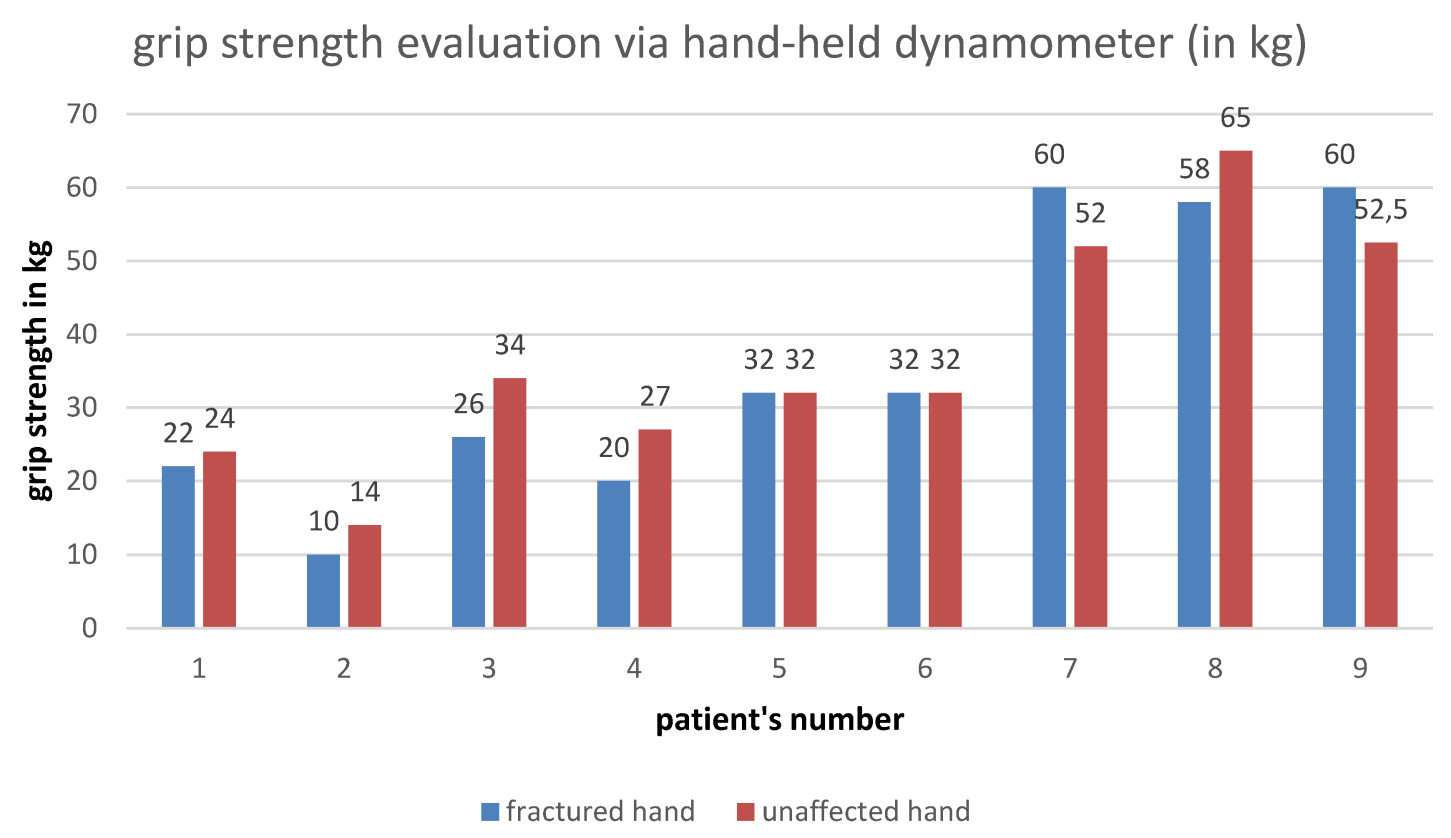
Grip strength evaluation of fractured versus unfractured hand

Grip strength evaluation was performed by using the hand-held dynamometer, visualized in Figure 5 (Fig. 5).

Eight of nine patients (88.89%) were satisfied with their overall outcome and stated full level of activity in the fields of work and physical activity. One patient with poorer outcome in long-term evaluation missed to return for removal of K-wire arthrodesis even one year after initial operation. This led to the high number in M²-DASH (52 points), seldom pain in rest, pain after mild activities of daily living, restricted flexion-extension arc, gap in opposition ability and pinch grip as well as less grip strength.

## Discussion

Fractures of the hamate, including hook fractures and those of the hamate body, are stated with 2–4% of all carpal fractures in the literature [1], [2], [3], [4], [5]. Concerning fracture-dislocations of the carpometacarpal region, the hamate-metacarpal fracture-dislocation constitutes 10–15% of those [11]. 

Generally, hamate fractures have to be subdivided into hook fractures and fractures of hamate’s body. Hook fractures were not discussed and included within this paper. Body fractures at all, are less frequent [19], [24]. This kind of fracture group consists of different entities with variant therapeutic considerations. The first classification of hamate fractures has been established by Milch et al. in 1934 [6]. It does not include the here discussed coronal fracture [6]. Due to further clinical and radiological investigations, other classifications were presented with a more detailed view on body fractures including coronal body fractures as well [7], [8], [9], [10]. Two of them are mostly named in literature – Cain’s classification on hamato-metacarpal-dislocations and Ebraheim’s classification on hamate fractures [7], [8]. Whereas Cain et al. concentrated on the dislocation of fifth CMC and further co-fractures of the hamate, Ebraheim et al. put the course of the fracture line through hamate’s body in the focus of classifying [7], [8].

For preoperative planning, we used Ebraheim’s variant of subdivision because we hypothesized, that this might deliver the best information necessary for treatment considerations. 

Some main facts about the specific anatomy and over all biomechanical situations in the healthy ulnar carpal region and its articulation with the metacarpal bones are necessary to know to understand the trauma leading to a fractured hamate bone. 

The precise use of hand functions is essential for everyday-life and a substantive factor of human beings. This evolutionary asset is realised through the concise interaction of different anatomical structures and bonds in the human hand. 

Sangole et al. postulated, that the kinematics of the CMC joints are necessary to let the palm form a bow and therefore realising a precise grip [29], [30]. The impairment of these CMC can lead to highly decreased grip strength [31]. 

El-Shennawy et al. performed a biomechanical study with special regard to the carpometacarpal joints (CMC) and their differences. The working group concluded that the degrees of movement are increasing from radial to ulnar. Therefore, the CMC V possesses the greatest range of motion (ROM) with special regard to the ROM of the fifth CMC depending on the unaffected movement of the fourth CMC [10], [32], [33]. The reason for this rise of mobility is the looser ligamentous attachments around CMC IV and V in comparison to those of CMC II and III [10], [34]. 

Fractures of the metacarpal bones IV and V as well as dislocations in the carpometacarpal joints can be accompanied by coronal fractures of the hamate [1], [2], [7], [8], [9], [10], [11], [12], [13], [14], [15], [17], [18], [19], [20], [21], [22], [23], [25], [26], [34], [35], [36], [37]. Therefore, a restricted mobility and function ensues [38], [39]. 

Coronal fractures of the hamate appear mostly after punching injuries or motor vehicle accidents [1], [4], [5], [7], [8], [10], [11], [12], [13], [14], [16], [17], [19], [20], [21], [23], [25], [26], [35], [37], [40], [41]. Other mechanisms of trauma are less frequent. Mainly, the fracture occurs after a transmission of forces along the fourth metacarpal (MC) axis [1], [5], [6], [7], [10], [11], [12], [14], [15], [20], [21], [24], [35], [36], [37], [42]. In a radial-deviated and about ten degree flexed fist, the distal part of the fourth MC bone stands upon the other anatomical structures and is the main point for loading the forces in a situation of falling or punching [11], [12]. Forwarding this kinetic energy leads to a fractured hamate and a shortening of the fourth finger including fourth metacarpal bone [7], [35]. This results in a loss of targeted power transmission towards the carpal structures and the base of fifth metacarpal. Now, the forces are directly transferred to the fifth MC shaft and may induce a fracture. Possible as well are base fractures of the fourth and fifth metacarpal bone [7], [21], [35]. Biomechanically important is the degree of flexion in CMC joint during the trauma; whereas a palmar-flexed MC bone is leading to a dorsal rim fracture of the hamate, a less flexed MC bone leads to the coronal body fracture and a furtherly extended MC bone contributes to a hook-fracture occurrence [2], [7], [35].

Typically, the coronal fracture of the body of the hamate imposes with a dorso-ulnar pain, accentuating by manual pressing or passive movement, dorsal ecchymosis and swelling [1], [2], [4], [5], [6], [7], [8], [11], [12], [13], [14], [15], [17], [18], [19], [20], [22], [26], [35], [37], [40], [43]. Further and more suspicious findings in clinical evaluation are caused by specific complications of the coronal fracture: palsy of motor branch of ulnar nerve, fractures of fourth and/or fifth MC, dislocations or sub-dislocations of CMC four and/or five, soft tissue damage, rupture of dorsal ligaments or rupture of extensor tendons. The compromising of ulnar nerve’s deep branch is either caused by contusion or by pressure induced by haemorrhage and/or oedema [1], [11], [20], [44], [45] and appears more often in connection with hook fractures because of its anatomical course around the ulnar side of the hamulus [1]. The impairment causes atrophy of interosseous muscles [11], [45]. 

Additionally, the coronal fracture of hamate’s body can be accompanied with either fractures of the fourth and/or fifth metacarpal bone or dislocations of the fourth and/or fifth CMC or a combination of both comorbidities. This causes a piano key phenomenon on the dorso-ulnar hand [11], [36] due to the mainly dorsally displaced MC base [1], [2], [7], [8], [10], [13], [14], [25], [35], [36], [43]. Furthermore, it leads to a weakened grip strength, limited opposition ability, pain accentuation by pressing along the MC axis and restricted mobility in CMC joints [6], [11], [12], [13], [14], [15]. The displacement is caused by a rupture of the interosseous ligaments that tie the metacarpals together. Other reasons are the rupture of the dorsal articular ligaments, the joint’s capsule, and the tensile forces of the tendons of flexor carpi ulnaris muscle and hypothenar muscles [2], [8], [10], [13], [20], [40], [46], [47], [48]. 

In most of our cases, the symptoms caused by accompanied injuries were predominant in comparison to those caused by hamate’s fracture itself. 

Without specific clinical findings caused by certain comorbidities or complications of hamate’s body fracture, the initial diagnosis is even more difficult. There is wide consent in literature, that the conventional x-ray images are incommensurate for diagnosing the coronal fracture of hamate’s body [2], [6], [7], [8], [11], [13], [14], [15], [16], [19], [20], [23], [24], [25], [26], [36]. Table 2 (Tab. 2) shows possible hints in x-ray images.

However, some authors described the initial x-rays without any obvious indications for a hamate’s fracture at all. This leads to an often-delayed diagnosis and therapy (either surgical or conservative) [2], [5], [16], [18], [20], [21], [23], [42], [49]. Langenhan et al. presented that even just one third of all coronal fractures of hamate’s body is found in initial presentation [11]. Chase et al. evaluated a diagnostic delay of one month after initial presentation and Ebraheim et al. an average delay of ten days [7], [20]. Wharton et al. performed a follow-up by data from the M-DASH and reported that one patient with later diagnosis and therefore delayed treatment had a significant less satisfying outcome than the others with lower delay in diagnosis [25]. This may be caused by the complications because of missing initial diagnose: muscular imbalance, arthritis, weakened grip strength and pseudarthrosis with persisting pain and functional impairment [2], [11], [14], [21], [24], [38], [42], [50]. It underlines the necessity for a clear algorithm in diagnosing hamate’s coronal fracture, to avoid these complications caused by delayed diagnostics. Gala et al. as well as Valente et al. are concerned that a strong clinical suspicion and proper radiological imaging are essential to recognize the fracture in patient’s initial presentation in the hospital [2], [23]. We want to add, that not only the clinical suspicion, but also the trauma history and the presence of co-fractures (like MC IV or V) can act as clues in initial diagnosing-process. In 2013 Gala et al. said, that the optimal and adequate radiological examination is still unclear [2]. Moreover, even nowadays there is no marked improvement. The standard radiographs (including lateral and anterior-posterior views) are mostly uncertain, as already outlined. As improvement, many authors gave the advice to conduct an oblique image of the carpal region [2], [4], [5], [7], [8], [11], [12], [13], [14], [17], [18], [20], [21], [24], [31], [37], [40]. 

However, the right angle seems to be unclear as well: there are different opinions whether 15°, 30° or 40–50° delivers the information needed [2], [7], [8], [14], [18], [24], [31], [40]. Andresen et al. published an evaluation in 1998 about hamate fractures (hook and corpus fractures included) in three different clinics over five years and they concluded, that in oblique views angling between 40 and 50 degrees, only 50% of all fractures were recognized [24]. Therefore they advised to perform ap, lateral and carpal tunnel (carpal tunnel view especially for hook fractures and very palmar body fractures) views standardly [24]. On the other hand, different authors recommend the 30° oblique view [2], [5], [8], [31], [40], whereas Cain et al. urge a combination of 15° and 45° supination image [7]. We have good results with a 30° oblique view, because of the fracture itself and the co-injuries getting unmasked clearly within this angle. However, we want to underline the special need for further studies dealing with the evaluation of the perfect angle of oblique x-ray images in carpal fractures.

Nonetheless, for further therapeutic planning, the performance of a computed tomography study is indispensable. Concerning this point, there is nearly unified consent in literature [2], [4], [5], [7], [8], [10], [11], [12], [13], [14], [15], [16], [17], [18], [19], [20], [21], [23], [24], [26], [35], [36]. Especially Andresen et al. gave objectified data in comparing x-ray’s and CT’s outcome in diagnosing coronal fractures of hamate bone: whereas the conventional x-ray studies have an accuracy of 80.5%, CT studies stated 97.2%. Furthermore, the CT is superior in sensitivity and specificity (100% and 94.4% compared to 72.7% and 88.8% for x-ray analysis) [24]. The data based on clinical findings and artificial fractured hamates in corpse hands [24]. 

In synopsis of all these findings and opinions concerning an adequate diagnostic process for coronal fracture of hamate’s body, we would like to recommend the following algorithm: Primarily, history with special regard to trauma mechanism and the clinical examination (with the knowledge of mostly unspecific clinical findings and the possible co-injuries) is necessary. The conventional x-ray analysis is supplemented with an additional 30° supination oblique image, to provide better visibility of CMC joint. A computer tomographic scan completes the diagnostic approach and forms the basis of further therapeutic planning. 

There is no clear treatment algorithm published in current literature. All advice given so far is based on experts’ opinions and either case reports or smaller case series with no statistical significance and plain evidence. Generally, there is a choice between a conservative or operative approach. The main point within this deliberation is, whether the coronal fracture of the hamate is supposed to be stable or unstable. By comparing the current literature, there is no clear consent towards this issue [2], [3], [8], [10], [11], [17]. Whereas authors like Busche et al. and Torres et al. described good results with their conservative treatment including a casting regimen, most of the other working groups decided for operative therapy in either open or closed reduction and internal fixation [1], [2], [9], [11], [12], [13], [14], [17], [18], [19], [20], [23], [37], [40], [41], [43], [51], [52]. Table 3 (Tab. 3) shows a compilation of studies dealing with coronal fractures and their therapy.

The conservative treatment can only be an option for coronal fractures without any dislocation at all. Gala et al. stated that this fracture type is exceedingly rare [2]. Additionally, in our case series, there were no isolated coronal fractures without any accompanied injuries. Kim et al. and Cain et al. support this observation with their finding that all CMC dislocations occur with any kind of hamate fracture are usually unstable and will dislocate again after initial closed reduction [7], [10]. Furthermore, Kang et al. saw the risk for an aggravated dislocation by just casting carpal fractures without any operative fixation [12]. Ebraheim et al. published a case series of 11 patients with hamate fractures combined with CMC dislocations. They treated 10 patients surgically and one case in closed reduction and casting. The loose of anatomical repositioning and the development of persistent subluxation in CMC were described in the conservative case [8]. Hence, the hamate fracture was considered unstable and required operative treatment to give best outcomes [8]. The results of our study support this conclusion. If the coronal hamate fracture is combined with further injuries, most of the results in previous literature underline the inadequacy of conservative treatment [2], [9], [11], [12], [15], [16], [20], [25], [35], [43]. Furthermore, we believe that subluxations in CMC are a strict contraindication for conservative treatment at all, due to the unstable situation and mostly poor outcomes in published studies [8], [11]. 

Reasons leading to the decision for operative treatment are mostly the dislocations in CMC joints, the instability of the fracture and included joint involvement [2], [7], [8], [9], [10], [11], [12], [13], [20], [25], [37], [43].

**Figure 6:**
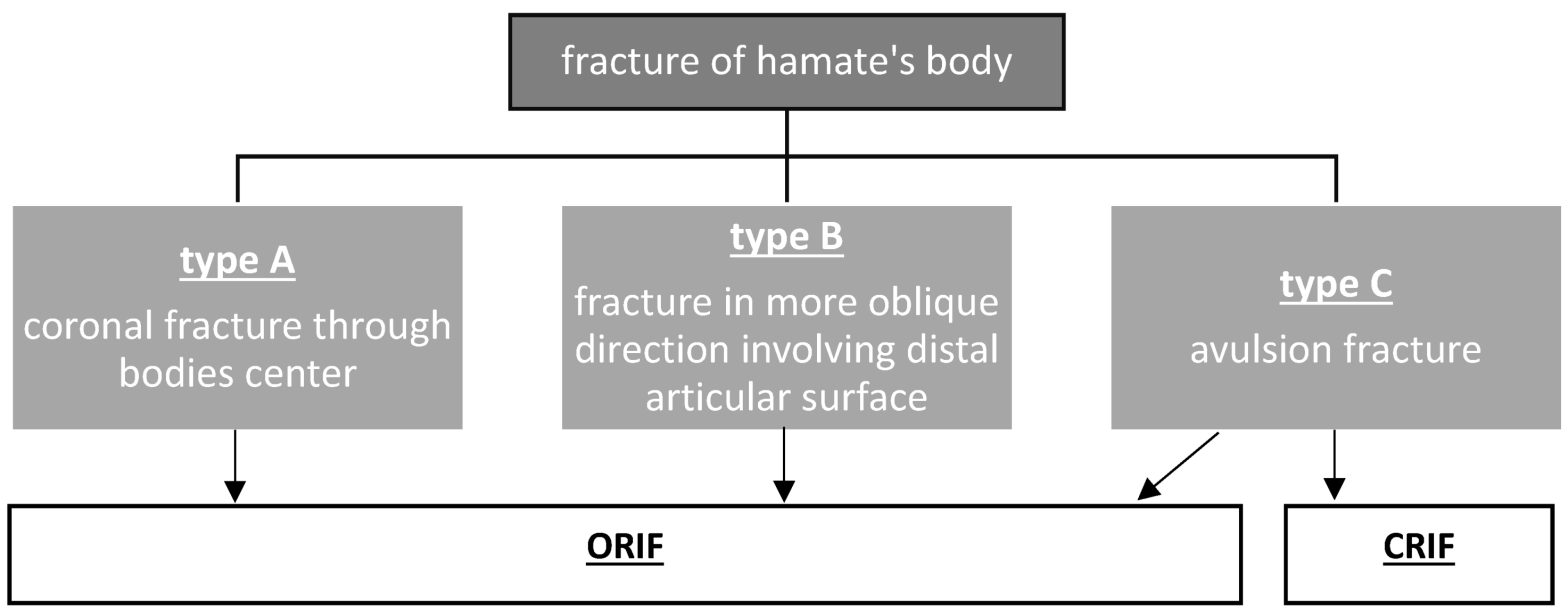
Ebraheim’s classification of coronal fractures of the hamate bone [8] with suggested treatment

The results of CRIF (closed reduction and internal fixation) against ORIF (open reduction and internal fixation) are difficult to compare because of the lack of larger case series or randomized trials. Additionally, even the indication for either the one or the other procedure is not defined clearly and inconclusive comparing different studies. Mainly, the decision for either ORIF or CRIF is influenced by the habitus of the fracture, visible in the CT scan preoperatively. If there are only a few fragments and the fracture dislocation is considered to be reconstructable with a closed approach including K-wire osteosynthesis, and there are no co-injuries requiring an open treatment, CRIF might be a valid option. In accordance to Ebraheim’s suggestions and his classification (Figure 6 (Fig. 6)), we decided for the following stage-depending treatment:

In type A and type B fractures an ORIF with either screw or plate osteosynthesis was performed. Closed reduction and internal fixation was chosen for type C fractures to reach anatomical reconstruction again [8].

The statements concerning follow-up examinations are very poor in current literature. Only Valente et al. described a functional impairment by evaluating deficiencies of 14° in flexing and 20° in extending CMC after CRIF [23], whereas nearly all of the other studies gave no further information to structured follow-up protocols [2], [8], [11], [12], [13], [16], [17], [18], [20], [40], [43]. Wharton et al. interviewed 12 of their patients by telephone with using the M-DASH score. They stated results reaching from 0 (3 cases) up to 45 (one case) points [25]. Two more cases where 35 and 36 points respectively with all the others laying in between. There is no average given and the reliability of follow-up stays restricted using the M2 DASH as subjective instrument for examination only [25]. The here used M² DASH uses a different point calculation, wherefore the comparability is unclear. Therefore, a detailed comparison of our long-term follow-up results with these of other study protocols is not possible at all. Kimura et al. gave their results of one case of coronal fracture treated with ORIF and K-wire osteosynthesis after ten months showing 70° flexion, 80° extension, 30° ulnar deviation, and 10° radial deviation [37]. The range of radial deviation is more restricted in their studies than in our examination in long-term follow-up, whereas the other movements are comparable to our findings.

Based on our own examinations, we hypothesize that there can be no clearly defined treatment algorithm for coronal hamate fractures at all. This is caused by a diversity of accompanied injuries, which demands for a high grade of experience to evaluate the situation of stability for each fracture individually. Plainly, in our opinion, there is no complete “stable” or “unstable” situation as hypothesized by some authors. Therefore, it has to be proven whether the fracture can be repositioned in a closed approach or if an open surgery is necessary. When performing ORIF, the surgeon has the choice between K-wire, screw, or plate osteosynthesis. As indicators for the different material serve the number of fragments, the extent of co-injuries, and their purport for stability in the ulnar carpal region and the expertise of the surgeon himself. 

Due to few case numbers, there was no option to perform an advanced subgroup analysis for comparing the outcome of these different material for osteosynthesis. Because of the rarity of hamate fractures in coronal plain, a multicenter and larger study has to be considered to generate reliable data for giving further advice. We can only say that the outcome of the patients treated and examined in long-term yields good results leading to the hypothesis that the different materials used are not the specific determining factors for functional outcome and patients’ satisfaction.

Limitations of this paper are the still small number of recruited patients and the amount of patient’s non-response. This furthermore leads to a small number of subgroups (e.g. plate versus screw in the ORIF group). Therefore, a significant analysis as well as valid and reliable outcomes comparing these subgroups are not feasible. There is specific need for subsequent investigations especially in randomized controlled groups to create more objective data. However, the here presented case series of coronal hamate fractures is, to our knowledge, the largest ever published. The clinical examination as well as the surgical therapy was performed by the same physician, which prevents interrater bias. The use of clearly defined algorithms in diagnostics and therapy avoid arbitrary decisions in single cases.

## Conclusion

In hamate fractures, the standard radiographs including an anterior-posterior and a lateral view only, are mostly inconclusive and incommensurate for the definite diagnosis and evaluation. Therefore, the diagnostic approach has to be completed with an oblique view of the carpal region and a CT scan, providing the basis for therapeutic planning. The conservative treatment is not to be recommended, due to high complication rates such as chronic subluxations in CMC joints and absent in pain relief. The open reduction and internal fixation, in comparison to the closed approach, offers the following advantages: anatomical repositioning of the fractured hamate bone, physiological restoration of articular surfaces, and possible remedying of co-injuries. The good postoperative results, in short- and long-term evaluation, are justifying the open approach. Especially the “add” case in the given list of cases shows the disadvantages of a conservative treatment in hamate’s coronal fracture. The closed reduction and internal fixation remain an option for type C body fractures (according to Ebraheim’s classification). Open reduction should always be considered intraoperatively as an operative extension.

## Notes

### Competing interests

The authors declare that they have no competing interests.

## Figures and Tables

**Table 1:**
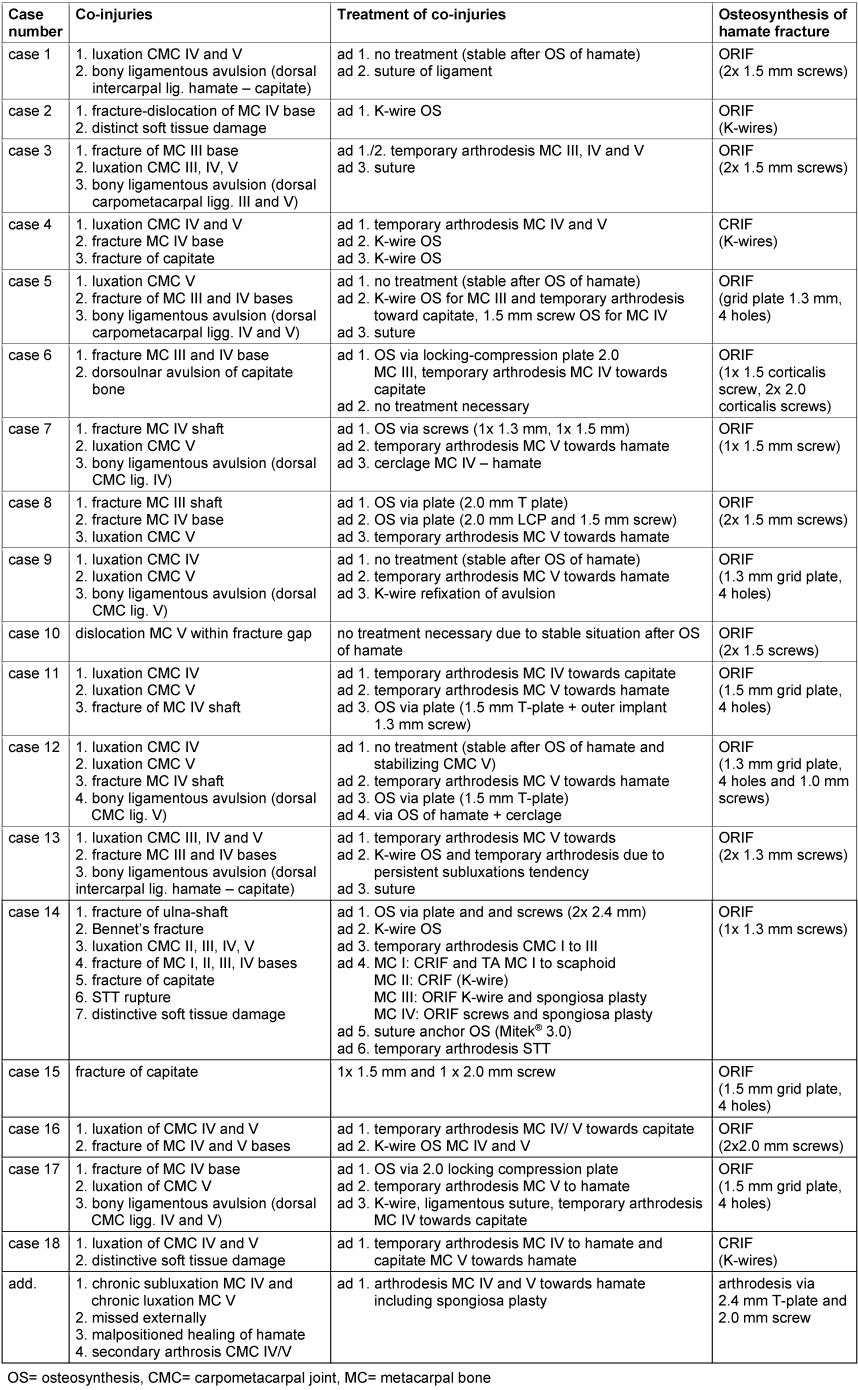
Listing of patient’s accompanied injuries, treatment of those and type of osteosynthesis material for hamate’s body

**Table 2:**
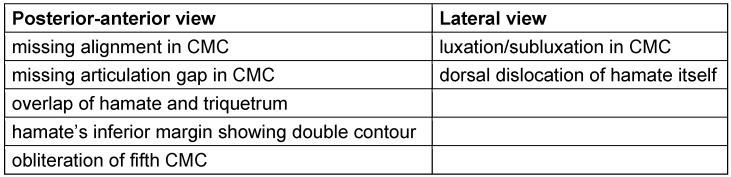
Hints for coronal hamate’s fracture in conventional x-ray images [1], [2], [8], [11], [20], [37]

**Table 3:**
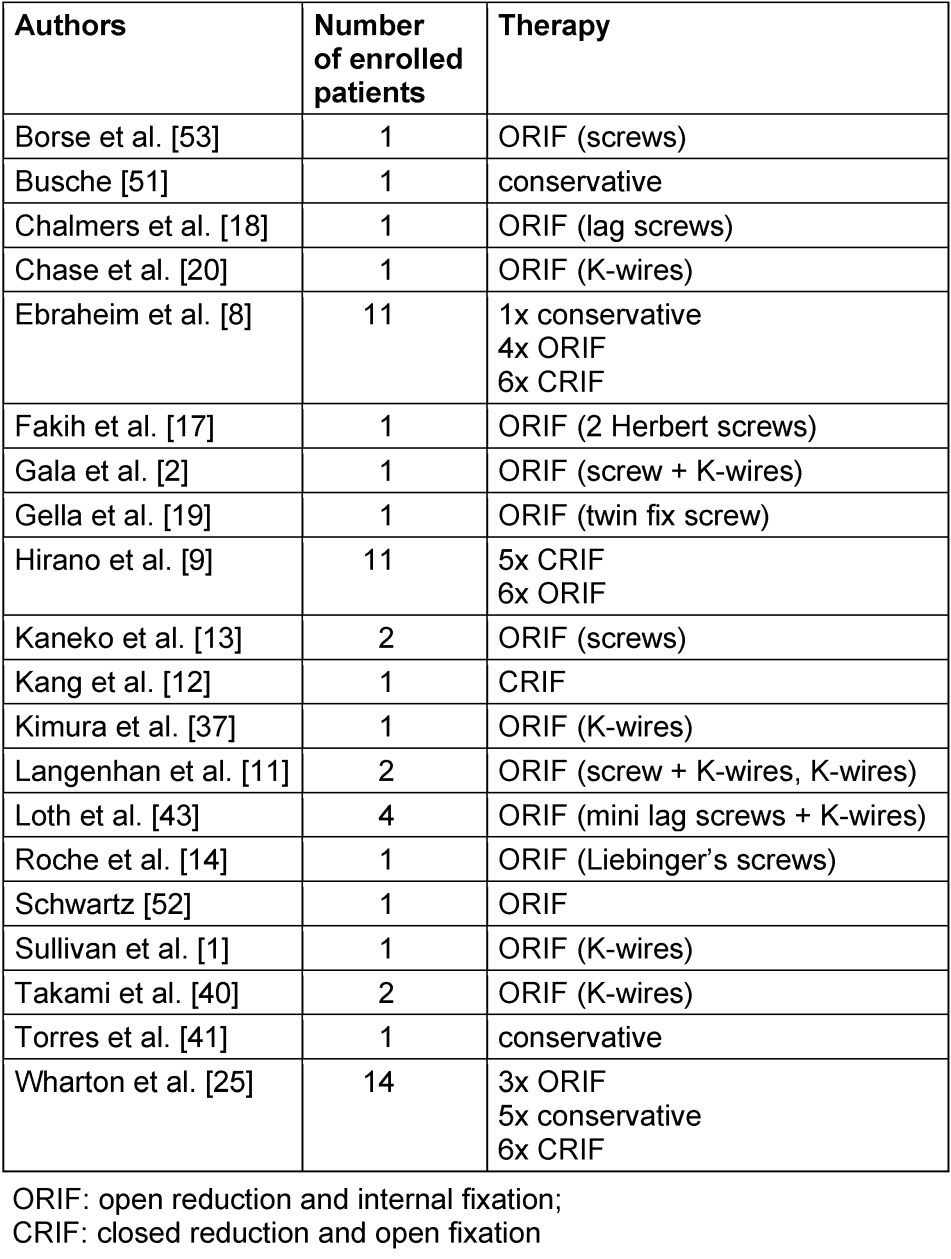
Collocation of studies dealing with coronal fractures of the hamate bone, showing patients count and chosen treatment.
